# Preoperative grading of intracranial meningioma by magnetic resonance spectroscopy (^1^H-MRS)

**DOI:** 10.1371/journal.pone.0207612

**Published:** 2018-11-19

**Authors:** Meng-Chi Lin, Chiao-Zhu Li, Chih-Chuan Hsieh, Kun-Ting Hong, Bon-Jour Lin, Chin Lin, Wen-Chiuan Tsai, Chiao-Hua Lee, Man-Gang Lee, Tzu-Tsao Chung, Chi-Tun Tang, Da-Tong Ju, Hsin-I Ma, Ming-Ying Liu, Yuan-Hao Chen, Dueng-Yuan Hueng

**Affiliations:** 1 Department of Neurological Surgery, Tri-Service General Hospital, National Defense Medical Center, Taipei, Taiwan; 2 Department of Surgery, Zuoying Branch, Kaohsiung Arm Force General Hospital, Kaohsiung, Taiwan; 3 Department of Surgery, Kaohsiung Arm Force General Hospital, Kaohsiung, Taiwan; 4 School of Public Health, National Defense Medical Center, Taipei, Taiwan, Republic of China; 5 Department of Pathology, Tri-Service General Hospital, National Defense Medical Center, Taipei, Taiwan; 6 Department of Radiology, Tri-Service General Hospital, National Defense Medical Center, Taipei, Taiwan; 7 Department of Biology and Anatomy, National Defense Medical Center, Taipei, Taiwan; 8 Department of Biochemistry, National Defense Medical Center, Taipei, Taiwan; 9 Graduate Institute of Medical Sciences, National Defense Medical Center, Taipei, Taiwan; National Research Council of Italy, ITALY

## Abstract

Although proton magnetic resonance spectroscopy (^1^H-MRS) is a common method for the evaluation of intracranial meningiomas, controversy exists regarding which parameter of ^1^H-MRS best predicts the histopathological grade of an intracranial meningioma. In this study, we evaluated the results of pre-operative ^1^H-MRS to identify predictive factors for high-grade intracranial meningioma. Thirteen patients with World Health Organization (WHO) grade II-III meningioma (confirmed by pathology) were defined as high-grade; twenty-two patients with WHO grade I meningioma were defined as low-grade. All patients were evaluated by ^1^H-MRS before surgery. The relationships between the ratios of metabolites (N-acetylaspartate [NAA], creatine [Cr], and choline [Cho]) and the diagnosis of high-grade meningioma were analyzed. According to Mann-Whitney U test analysis, the Cho/NAA ratio in cases of high-grade meningioma was significantly higher than in cases of low-grade meningioma (6.34 ± 7.90 vs. 1.58 ± 0.77, p<0.05); however, there were no differences in age, Cho/Cr, or NAA/Cr. According to conditional inference tree analysis, the optimal cut-off point for the Cho/NAA ration between high-grade and low-grade meningioma was 2.409 (sensitivity = 61.54%; specificity = 86.36%). This analysis of pre-operative ^1^H-MRS metabolite ratio demonstrated that the Cho/NAA ratio may provide a simple and practical predictive value for high-grade intracranial meningiomas, and may aid neurosurgeons in efforts to design an appropriate surgical plan and treatment strategy before surgery.

## Introduction

Because of the aggressive behavior and poor prognosis of high-grade intracranial meningiomas, neurosurgeons must preoperatively distinguish high-grade and low-grade intracranial meningiomas; however, in some cases, these tumors may not readily be differentiated by conventional magnetic resonance imaging (MRI). Thus, surgeons may obtain more information regarding intracranial tumors through the use of proton magnetic resonance spectroscopy (^1^H-MRS), a non-invasive, quantitative technique for estimating metabolite ‬ inside the ‬tumor. These metabolites include N-acetylaspartate (NAA), creatine (Cr), and choline (Cho), which have been used to predict the histopathological grade of cerebral glioma[1–5]. However, to the best of our knowledge, there has been no consensus regarding which of them might reliably predict high-grade intracranial meningiomas. ‬‬‬‬‬‬‬‬‬‬‬‬‬‬‬‬‬‬‬‬‬‬‬‬‬‬‬‬‬‬‬‬‬‬‬

Thus, this study aimed to evaluate whether the results of preoperative ^1^H-MRS correlated with the diagnosis of high-grade meningiomas.

## Materials and methods

### Clinical data

This study was assessed as low risk, therefore, the Institutional Review Board of the Tri-Service General Hospital approved this study (TSGHIRB No.: 2-106-05-011) as above-named application for signature exemption on informed consent form. In this retrospective study, from July 2008 to May 2016, 70 patients with intracranial meningioma were collected and studied in our hospital, a tertiary health center. A total of 35 patients were analyzed in this study; the remaining 35 patients were excluded due to history of radiotherapy, chemotherapy, or transarterial embolization before surgery, recurrent intracranial meningiomas, and/or inadequate preoperative MRI and ^1^H-MRS examinations. The 35 patients (16 male and 19 female; age range, 20–86 years old; mean age, 58.8 years old) (Table 1) that were included in this study were postoperatively diagnosed with primary intracranial meningioma by pathological findings. Based on the 2007 World Health Organization (WHO) criteria, 22 patients exhibited grade I meningiomas, 10 patients exhibited grade II meningiomas and three patients exhibited grade III meningiomas.

**Table 1:** Characteristics of the study groups.

Variable	Number of Patients	(%)
**Gender**		
**Female**	19	54.3
**Male**	16	45.7
**Grade**^**a**^		
**High**	13	37.1
**Low**	22	62.9

^a^High-grade: WHO grade II-III; Low-grade: WHO grade I

### MRI protocol

Before surgery, each patient received MRI and ^1^H-MRS evaluation with 1.5-Tesla (1.5-T) MRI scanner (Vision Plus, Siemens, Germany). The MRI sequence was executed with a repetition time (TR) of 2140 msec, echo time (TE) of 35 msec, inversion-time (TI) of 420 msec, matrix size of 256 × 256, slice thickness of 3 mm, and intersection gap of 0.2 mm. The brain imaging protocol comprised T1-weighted spin-echo (SE) imaging, T2-weighted SE imaging, and fluid-attenuated inversion recovery (FLAIR) imaging. Gadolinium-enhanced T1-weighted spin-echo SE imaging was acquired after intravenous administration of contrast (0.1 mmol of gadolinium per kg of body weight).

### Spectroscopy

**Fig 1:**
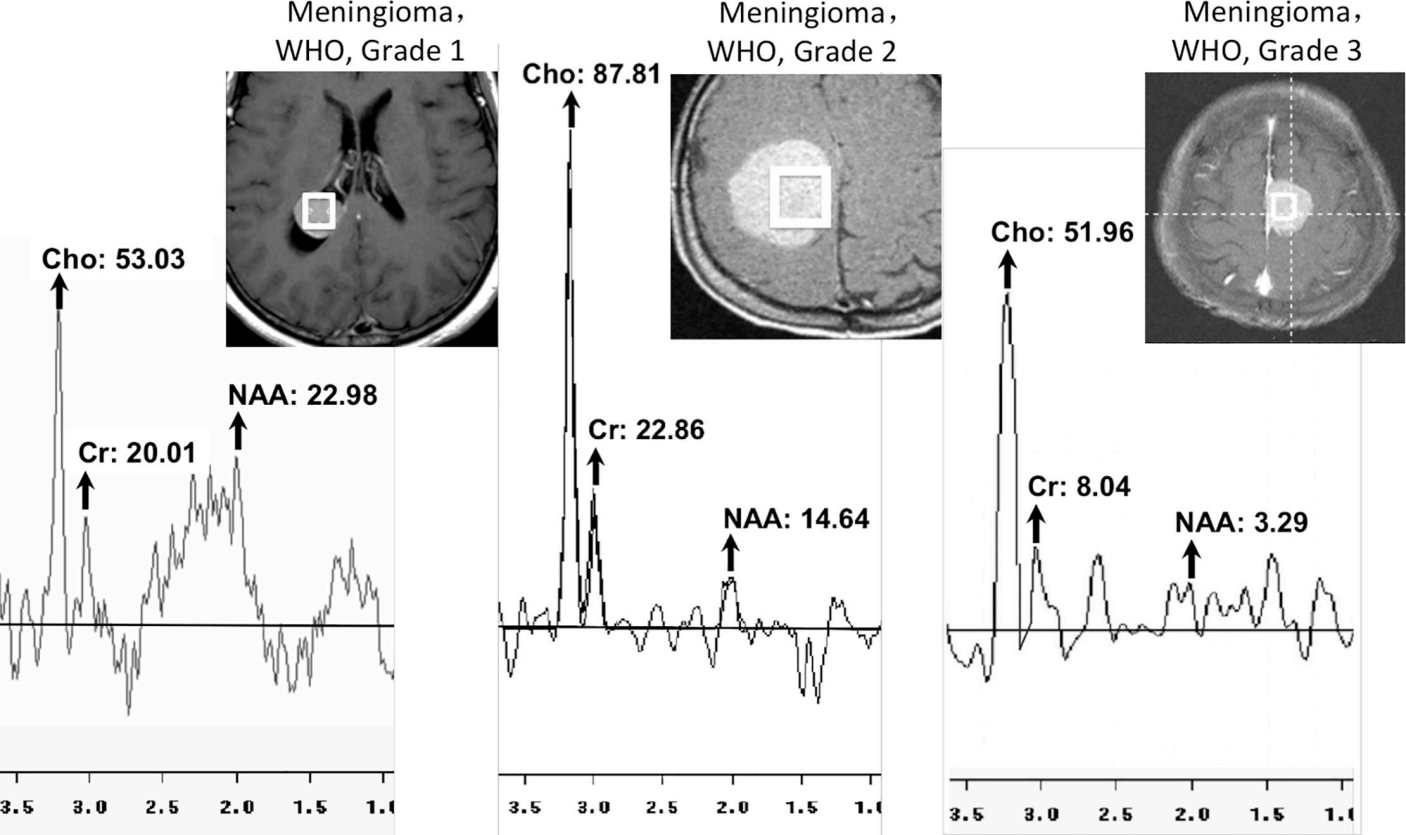
**Left, Spectrum of meningioma, WHO, grade 1; Middle, Spectrum of meningioma, WHO, grade 2; Right, Spectrum of meningioma, WHO, grade 3.** Cho, choline; Cr, creatine; NAA, N-acetylaspartate.

MR spectroscopy (Fig 1) was performed via a single-volume, stimulated-echo acquisition mode with a TE of 35 ms. To avoid the influence of skull, fat, cyst, necrosis, edema, hemorrhage and calcification, the spectroscopic volume of interest (VOI) was adjusted carefully and placed as much as possible within the range of intracranial tumors, which were visualized by previous three-dimensional gadolinium-enhanced T1-weighted imaging. The size of the VOI was designated as between 1.5 × 1.5 × 1.5 cm^3^ (3.4 mL) and 2 × 2 × 2 cm^3^ (8 mL). Based on the spectroscopic results of intracranial meningiomas, the concentrations of metabolites, including N-acetylaspartate (NAA) [2.0 ppm], creatine (Cr) [3.0 ppm] and choline (Cho) [3.2 ppm], were calculated, and the following metabolite ratios were determined: Cho/Cr, Cho/NAA, NAA/Cr.

### Pathological confirmation

A total of 35 paraffin-embedded tissues, resected from meningiomas, were reviewed by two experienced pathologists, who confirmed the pathological diagnosis based on the criteria within the 2007 WHO classification of meningiomas. The definition of grade II meningioma was based on one of the following criteria: 1) brain invasion on tumor histology, 2) 4–19 mitoses per 10 high-power fields, or 3) ≥3 of the following tumor features: sheet-like growth, increased cellularity, small cells, prominent nucleoli, or necrosis. The grade III meningioma was based on one of the following criteria: 1) ≥ 20 mitoses per 10 high-power fields, or 2) the presence of malignant cytology resembling carcinomas, melanomas, or high-grade sarcomas.

### Statistical analysis

All data were collected and analyzed via SPSS (R v3.3.0) statistical program; *p* values < 0.05 were considered to be statistically significant. The correlations between Cho/Cr, Cho/NAA, NAA/Cr, and the histopathological grade of intracranial meningiomas were assessed via Mann-Whitney U tests. A conditional inference tree was applied to determine the optimal cut-off point to predict high-grade intracranial meningiomas.

## Results

**Fig 2:**
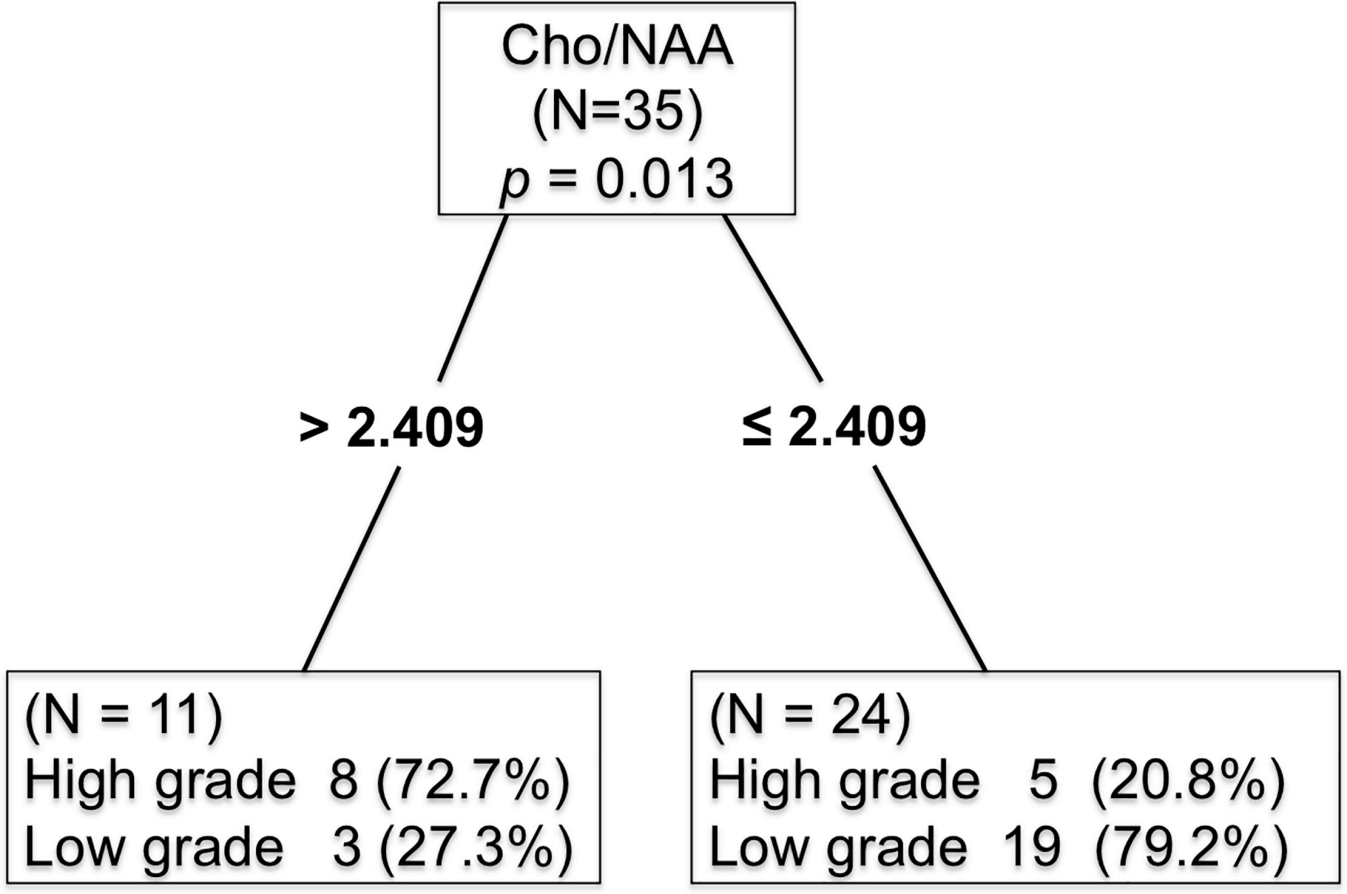
Conditional inference tree revealing that the optimal cut-off point to distinguish high-grade meningioma from low-grade meningioma was 2.409 (OR = 10.133). Among 13 high-grade meningiomas, eight exhibited a Cho/NAA ratio of >2.409, sensitivity = 61.54%; among 22 low-grade meningiomas, 19 exhibited a Cho/NAA ratio of ≤2.409, specificity = 86.36%. For this cut-off value, the positive predictive value (PPV) was 72.7%, whereas the negative predictive value (NPV) was 79.2%. Cho, choline; Cr, creatine; NAA, N-acetylaspartate.

Our study included 35 patients: 13 exhibited high-grade meningiomas (10 that were WHO grade II and three that were WHO grade III) and 22 exhibited low-grade meningiomas (WHO grade I); detailed patient characteristics are shown as Table 1. The outcome of ^1^H-MRS metabolite analysis is shown in Table 2. The respective mean values of Cho/Cr, Cho/NAA and NAA/Cr ratios were 2.69 ± 2.19, 6.34 ± 7.90, and 1.01 ± 0.76 (mean ± standard deviation) for high-grade meningiomas; these same respective ratios were 2.50 ± 2.48, 1.58 ± 0.77, and 1.71 ± 1.37 for low-grade meningiomas. Detailed results are provided in Table 3. Among these ratios, only Cho/NAA was significantly different between high-grade and low-grade meningiomas (*p* = 0.013); Cho/Cr and NAA/Cr were not significantly different (*p* = 0.578; *p* = 0.052). We used the conditional inference tree to predict the probability of high-grade meningioma via analyses of Cho/NAA, Cho/Cr, and NAA/Cr. The most predictor of highest importance was Cho/NAA; thus, all patients were classified into two groups according to this variable, and the optimal cut-off point to distinguish high-grade meningioma from low-grade meningioma was revealed as 2.409 (OR = 10.133), shown in Fig 2. Among 13 high-grade meningiomas, eight exhibited a Cho/NAA ratio of >2.409, sensitivity = 61.54%; among 22 low-grade meningiomas, 19 exhibited a Cho/NAA ratio of ≤2.409, specificity = 86.36%. For this cut-off value, the positive predictive value (PPV) was 72.7%, whereas the negative predictive value (NPV) was 79.2%.

**Table 2:** Proton magnetic resonance spectroscopy (^1^H-MRS)-detected metabolite ratios in intracranial meningioma.

Variable	Cho/Cr	Cho/NAA	NAA/Cr
**Min**	0.380	0.352	0.020
**Mean**	2.568	3.346	1.451
**Median**	1.695	1.700	1.177
**Max**	12.340	27.130	6.990

Cho choline, Cr creatine, NAA N-acetylaspartate

**Table 3:** Univariate analysis of potential predictors for high-grade meningioma.

Variable	High-grade^a^	Low-grade^a^	*p* value
**Age**	58.23 ± 20.29	59.18 ± 15.11	0.986
**Gender**			
**Female**	12 (54.5%)	7 (53.8%)	
**Male**	10 (45.5%)	6 (46.2%)	
**Cho/Cr**	2.69 ± 2.19	2.50 ± 2.48	0.578
**Cho/NAA**	6.34 ± 7.90	1.58 ± 0.77	0.013^b^
**NAA/Cr**	1.01 ± 0.76	1.71 ± 1.37	0.052

^a^High-grade: WHO grade II-III; Low-grade: WHO grade I

^b^*p* < 0.05, statistically significant

(Testing by Fisher exact test, Wilcoxon Test, or Kruskal-Wallis Test, respectively. Continuous variate data are presented as Mean ± STDEV.)

## Discussion

Preoperatively estimating the histopathological grade of intracranial meningioma accurately is very important when establishing an appropriate treatment plan. Many studies [6–8] have reported that patients with high-grade meningiomas exhibit poorer outcome and higher recurrence rates than patients with low-grade meningiomas. When preoperative examinations indicate a high-grade meningioma, an aggressive treatment plan should be arranged, even if the tumor is small and the patient is advanced in age. In contrast, when a low-grade intracranial meningioma is found, follow-up without surgery sometimes may be appropriate for the patient.

Shino et al. [9] reported that the Cho/Cr ratio can serve as a useful predictive parameter to assess the malignant potential of meningiomas. However, they did not measure NAA concentration in the study; moreover, preoperative embolization was performed in seven patients in order to reduce intraoperative blood loss, which may have influenced intratumor metabolism. Nevertheless, this study revealed that the metabolites detected by ^1^H-MRS could predict the malignant potential of meningiomas.

Majos et al. [10] demonstrated that the ^1^H-MRS of high-grade meningiomas exhibited the trend of the metabolites as increasing concentrations of Cho while decreasing concentrations of NAA. Consistently, their study further supported our results.

In this study, we classified WHO grade II-III meningiomas as high-grade, and WHO grade I meningiomas as low-grade. After analyzing the ratios of Cho/Cr, Cho/NAA, and NAA/Cr between these two grades, we found that Cho/NAA exhibited significant differences between high-grade and low-grade meningiomas (*p* = 0.013). In addition, we found that the optimal cut-off point to distinguish high-grade meningioma from low-grade was 2.409 (OR = 10.133), which resulted in sensitivity of 61.54%, specificity of 86.36%, PPV of 72.7%, and NPV of 79.2%.

Kawahara et al. [11] proposed that the combination of an unclear tumor-brain interface on T1-weighted imaging and a heterogeneous enhancement on MR imaging could be useful to predict high-grade meningioma. Lin et al. [12] also designed a prediction model for the pathological grading of meningioma, consisting of a scoring scale: 2 × (age) + 5 × (tumor-brain interface) + 3 × (capsular enhancement) + 2 × (tumor enhancement). However, Shino et al. [9] reported that some high-grade meningiomas may exhibit similar MR imaging findings to low-grade meningiomas. Furthermore, some subjective bias may exist in the interpretation of imaging findings.

This study aimed to identify a simple and quantitative parameter that could help clinicians predict high-grade meningiomas easily, thereby facilitating the design of an appropriate treatment plan. Thus far, no unified guideline has existed to indicate which parameter could best predict the histopathological grade of meningioma.

In our study, we found that the Cho/NAA ratio, which is easily and widely obtained during ^1^H-MRS, may predict high-grade meningiomas when its value is >2.409 (sensitivity = 61.54%; specificity = 86.36%). This simple and quantitative parameter may help neurosurgeons to select either a more aggressive treatment strategy in cases with higher likelihood of high-grade meningioma, or a conservative approach in cases of old and/or asymptomatic patients with higher likelihood of low-grade meningioma.

Jaskólski [13] reported that MRS could not distinguish between high-grade and low-grade meningiomas. However, their study only included 14 cases of meningiomas (five high-grade; nine low-grade). In our study, we collected total 35 meningiomas (13 high-grade; 22 low-grade), which may thus provide more effective information regarding the correlation between MRS and the grading of meningiomas.

There are some limitations in our study. First, all patients came from single tertiary health center; the patient number was relatively small. Second, this study was retrospective in nature; further large-population, multi-center, prospective studies may be needed to assess the accuracy of our cut-off point. Finally, the method of calculating the Cho/NAA ratio may provide some cause for concern; however, we assumed that its influence on high-grade meningiomas ought to be similar to its influence on low-grade meningiomas, and would therefore not affect the results of our study.

## Conclusions

This analysis of preoperative ^1^H-MRS metabolite ratios found that Cho/NAA may serve as a simple and practical predictive value for high-grade intracranial meningiomas, and may help neurosurgeons to design an appropriate surgical plan and treatment strategy prior to surgery.
